# Transcriptome profiling of TDC cluster deletion mutant of *Enterococcus faecalis* V583

**DOI:** 10.1016/j.gdata.2016.06.012

**Published:** 2016-06-23

**Authors:** Marta Perez, Victor Ladero, Beatriz del Rio, Begoña Redruello, Anne de Jong, Oscar P. Kuipers, Jan Kok, M. Cruz Martin, Maria Fernandez, Miguel A. Alvarez

**Affiliations:** a Instituto de Productos Lácteos de Asturias, IPLA-CSIC, Paseo Rio Linares s/n, 33300, Villaviciosa, Spain; b Department of Molecular Genetics, Groningen Biomolecular Sciences and Biotechnology Institute, University of Groningen, Nijenborgh 7, 9747 AG Groningen, The Netherlands

**Keywords:** *Enterococcus faecalis*, Biogenic amines, Tyramine, Tyrosine decarboxylase cluster, Microarray

## Abstract

The species *Enterococcus faecalis* is able to catabolise the amino acid tyrosine into the biogenic amine tyramine by the tyrosine decarboxilase (TDC) pathway Ladero et al. (2012) [1]. The TDC cluster comprises four genes: *tyrS*, an aminoacyl-tRNA synthetase-like gene; *tdcA*, which encodes the tyrosine decarboxylase; *tyrP*, a tyrosine/tyramine exchanger gene and *nhaC*-*2*, which encodes an Na^+^/H^+^ antiporter and whose role in the tyramine biosynthesis remains unknown [2]. In *E. faecalis* V583 the last three genes are co-transcribed as a single polycistronic mRNA forming the catabolic operon, while *tyrS* is transcribed independently of the catabolic genes as a monocistronic mRNA [2]. The catabolic operon is transcriptionally induced by tyrosine and acidic pH. On the opposite, the *tyrS* expression is repressed by tyrosine concentrations [2]. In this work we report the transcriptional profiling of the TDC cluster deletion mutant (*E. faecalis* V583 ΔTDC) [2] compared to the wild-type strain, both grown in M17 medium supplemented with tyrosine. The transcriptional profile data of TDC cluster-regulated genes were deposited in the Gene Expression Omnibus (GEO) database under accession no. GSE77864.

**Table:** 

Specifications	
Organism/cell line/tissue	*E. faecalis* V583
Sex	N/A
Sequencer or array type	Oligo-based DNA microarray
Data format	Raw and normalized
Experimental factors	*E. faecalis* V583 ΔTDC (test) versus *E. faecalis* V583 (reference)
Experimental features	Microarray comparison was preformed to identify genes differentially expressed in *E. faecalis* V583 ΔTDC compared to *E. faecalis* V583 grown in M17 medium supplemented with 0.5% glucose (w/v) and 15 mM tyrosine.
Consent	N/A
Sample source location	Villaviciosa, Spain

## 1 Direct link to deposited data

Microarray data are accessible in the link: http://www.ncbi.nlm.nih.gov/geo/query/acc.cgi?acc=GSE77864

## 2 Experimental design, materials and methods

### 2.1 Design of *E. faecalis* V583 DNA microarrays

*E. faecalis V583* DNA microarrays (Agilent Technologies, Santa Clara, CA) were designed using the Agilent eArray (v5.0) program according to the manufacturer's recommendations. Each microarray (8 × 15 K) was designed to contain spots of two different 60-mer oligonucleotide probes (in duplicate) specific for each of the 3182 coding DNA sequences (CDSs) representing the chromosomal genes of the *E. faecalis* V583 genome (GenBank accession no. AE016830) [3].

### 2.2 Bacterial strains and growth conditions

*E. faecalis* V583 is a human clinical isolate [4]. The non-tyramine-producing mutant *E. faecalis* V583 ΔTDC was previously constructed by double-crossover homologous recombination [2]. Both strains were grown in 30 ml of M17 culture medium (Oxoid, Basingstoke, United Kingdom) supplemented with 0.5% glucose (w/v) (GM17) and 15 mM tyrosine (Sigma-Aldrich, Barcelona, Spain) for 5 h at 37 °C without aeration. After a centrifugation at 16,000 ×* g* for 1 min at 4 °C, the supernatants were discarded and cell pellets were frozen in liquid nitrogen and stored at − 80 °C.

### 2.3 RNA extraction

RNA isolation was performed as previously described [5] with minor modifications. Briefly, cell pellets were resuspended in 400 μl of TE buffer (10 mM Tris-HCl, 1 mM EDTA pH 8.0) and 50 μl of 10% SDS, 500 μl of phenol:chloroform:isoamyl alcohol (25:24:1) (Sigma-Aldrich), 500 mg of glass beads (75–150 μm) (Sigma-Aldrich), and 175 μl of Macaloid suspension (Bentone MA, Rheox Inc., Scotland, United Kingdom) were added. Cells were mechanically disrupted in a bead beater at 4 °C with two cycles of 60 s. During the shaking intervals the cells were kept on ice for 1 min. The samples were then centrifuged at 16,000 ×* g* for 1 min at 4 °C. The upper phase was transferred to fresh tubes containing 500 μl chloroform: isoamyl alcohol (24:1) and centrifuged for 5 min at 4 °C. 500 μl of the upper phase were transferred to fresh tubes and total RNA was isolated with the High Pure RNA Isolation Kit (Roche Diagnostics GmbH, Mannheim, Germany) following the instructions provided by the manufacturer. The concentration and quality of the RNA was determined on a NanoDrop spectrophotometer (Thermo Scientific, Landsmeer, The Netherlands).

### 2.4 Synthesis of cDNA

Total cDNA was synthesized in 30 μl volume reaction from 20 μg of RNA using the SuperScript® III Reverse Transcriptase kit (Life Technologies, Bleiswijk, Netherlands) as previously described [5]. The mRNA of the reverse transcription mixture was denaturalized by adding 3 μl of 2.5 mM NaOH for 15 min at 37 °C and then it was neutralized by adding 15 μl of 2 M HEPES free acid. The cDNA was purified using the NucleoSpin Gel and PCR Clean-up kit (Macherey-Nager, Landsmeer, The Netherlands) following the manufacturer instructions. Briefly, 200 μl of NTC buffer were mixed with the unpurified cDNA, added to a column and centrifuged for 1 min at 11,000 ×* g*. The column was washed first with 600 μl of buffer NT3 and then with 500 μl 80% ethanol. The residual ethanol was completely removed by centrifugation for 2 min at 11,000 ×* g*. Elution of the cDNA was done by addition of 60 μl of 0.1 M sodium bicarbonate pH 9.0 to the column and incubation for 1 min at room temperature. Purified cDNA was collected by centrifugation for 1 min at 11,000 ×* g* and was immediately labelled.

### 2.5 Labelling of cDNA

Twenty μg of the purified cDNA was labelled with DyLight 550 or DyLight 650 using the DyLight® Amine-Reactive Dyes kit (Thermo Scientific) as previously described [6]. Labelled cDNA was purified using NucleoSpin Gel and PCR Clean-up columns as described above, with the exception that cDNA was eluted with 50 μl of elution buffer NE of the NucleoSpin Gel and PCR Clean-up kit. Quality and quantity of cDNA and DyLight 550 and DyLight 650 labelling were checked on a NanoDrop spectrophotometer (Thermo Scientific, Landsmeer, The Netherlands).

### 2.6 Hybridization and washing

Three hundred ng of DyLight 550- and three hundred ng of DyLight 650-labelled cDNA were mixed and hybridized for 17 h at 60 °C in the *E. faecalis* V583 DNA microarray using the In situ Hybridization Kit Plus, the Hybridization Gasket Slide and the Agilent G2534 A Microarray Hybridization Chamber (Agilent Technologies). Slides were washed using the washing buffers indicated by the manufacturer.

### 2.7 Microarray data analysis

Slides were scanned using a GenePix 4200 A Microarray Scanner (Molecular Devices, Sunnyvale, CA). Slide images were analysed using GenePix Pro v.6.0 software. Background subtraction and LOWESS (locally weighted scatterplot smoothing) normalization were done using the standard routines provided by GENOME2D software available at http://genome2d.molgenrug.nl/index.php/analysis-pipeline. DNA microarray data were obtained from two independent biological replicates and one technical replicate (including a dye swap). Expression ratios were calculated from the comparison of four spots per gene per microarray (total of 20 measurements per gene). A gene was considered differentially expressed when a *p* value of at least < 0.05 was obtained and the expression fold-change was at least >|2 |. The microarray data were deposited in Gene Expression Omnibus (GEO) database under the Accession no. GSE77864.

## 3 Discussion

In the present work, we studied the effect of TDC cluster deletion on the transcriptomic profile of *E. faecalis* V583 grown in M17 supplemented with 0.5% glucose and 15 mM tyrosine. The expression of the deleted genes *tyrS*, *tdcA* and *tyrP* was reduced in the mutant strain. However, differences on the expression of *tyrP* gene between the *Δ*TDC mutant and V583 wild-type strain were not statistically significant, which suggests a low expression of *tyrP* on the wild-type strain. Unexpectedly, the *nhaC*-*2* gene was overexpressed in the ΔTDC mutant strain. This result can be explained taking into account (i) how the mutant strain was constructed [2], that is keeping the 5 ‘end of the first gene of the cluster (*tyrS*) and the 3’ end of the last one, which is *nhaC*-*2*, and (ii) one of the two *nhaC*-*2* gene probes designed for the array hybridizes with the 3′ remaining region of *nhaC*-*2*. Thus a polar effect would cause the *nhaC*-*2* overexpression in the *Δ*TDC mutant.

Additionally, other 18 genes were downregulated and 7 upregulated in the ΔTDC mutant strain compared to the V583 wild-type strain. Most of them were involved in transport and metabolism of amino acids. These results agree with the reduced production of ABC transporters in *E. faecalis* when it is grown in culture media supplemented with tyrosine [7]. In addition, tyrosine seems to inhibit pathways involved in the biosynthesis of aromatic compounds in *E. faecalis* [7]. Accordingly, the *aroE* gene (*EF1561*) involved in the biosynthetic routes of aromatic amino acids, including tyrosine, was also downregulated in the ΔTDC mutant strain. Further investigations will be required to elucidate the role of TDC cluster in the functions of the other regulated genes.
